# Acquired Resistance to Immune Checkpoint Blockade Therapies

**DOI:** 10.3390/cancers12051161

**Published:** 2020-05-05

**Authors:** Xianda Zhao, Dechen Wangmo, Matthew Robertson, Subbaya Subramanian

**Affiliations:** 1 Department of Surgery, University of Minnesota Medical School, Minneapolis, MN 55455, USA; zhaox714@umn.edu (X.Z.); wangm005@umn.edu (D.W.); robe1414@umn.edu (M.R.); 2 Masonic Cancer Center, University of Minnesota, Minneapolis, MN 55455, USA; 3 Center for Immunology, University of Minnesota, Minneapolis, MN 55455, USA

**Keywords:** tumor immunology, immune checkpoint blockade, immune response, acquired resistance, CTLA-4, PD-1, T cells

## Abstract

Immune checkpoint blockade therapy (ICBT) has revolutionized the treatment and management of numerous cancers, yet a substantial proportion of patients who initially respond to ICBT subsequently develop resistance. Comprehensive genomic analysis of samples from recent clinical trials and pre-clinical investigation in mouse models of cancer provide insight into how tumors evade ICBT after an initial response to treatment. Here, we summarize our current knowledge on the development of acquired ICBT resistance, by examining the mechanisms related to tumor-intrinsic properties, T-cell function, and tumor-immune cell interactions. We discuss current and future management of ICBT resistance, and consider crucial questions remaining in this field of acquired resistance to immune checkpoint blockade therapies.

## 1. Introduction

Therapeutic strategies which utilize patients’ immune systems to fight cancer have been investigated for over 100 years, beginning with Dr. Wilhelm Busch who infected patients with erysipelas (bacterial skin infection) and observed tumor regression [1,2]. However, early iterations of cancer immunotherapies, such as tumor vaccines and cytokine-based treatments, showed only moderate efficacy in a few cancer types [3]. These attempts were unsuccessful because they lacked specificity to antitumor immune regulations. Nonspecific enhancement of immune mechanisms inevitably leads to highly toxic side effects, which limits the efficacy and narrows the indications of these therapies. As our understanding of antitumor immunity has expanded in recent years, immune checkpoint blockade therapies (ICBT) targeting key regulators of antitumor immunity have been successfully tested. ICBT, such as anti-programmed cell death protein 1 (anti-PD-1)/anti-programmed death-ligand 1 (anti-PD-L1) and anti-cytotoxic T-lymphocyte-associated protein 4 (anti-CTLA-4) now represent a new class of cancer therapeutics.

Tumors are rich sources of neoantigens and thus, are potently immunogenic. Immune checkpoints, which provide costimulatory and coinhibitory signals to either boost or restrict T-cell immune responses, are the major players in the manipulation of the antitumor immune response. First-generation ICBT primarily targets the CD28/CTLA-4 and the PD-1/PD-L1 signaling pathways, to revitalize functionally suppressed T cells in tumor conditions (detailed mechanisms were summarized in the previous reviews) [4,5]. The widespread use of ICBT began in 2011 with the FDA approval of Ipilimumab, an anti-CTLA-4 treatment for advanced melanoma patients. Since then, ICBT has been successfully tested as a first- or second-line treatment for lung, kidney, head and neck, bladder, liver, stomach, colon, and other cancers [6].

However, like all other cancer treatments, the efficacy of ICBT is limited by both intrinsic and acquired resistance. Intrinsic resistance is defined as either tumor progression or no response upon initial administration of ICBT [7]. In contrast, acquired resistance develops in patients who initially showed encouraging signs of tumor regression. Acquired resistance also impairs the duration of clinical benefit. Although the exact incidence of acquired ICBT resistance is not yet well documented, it is clear that acquired resistance develops in a subset of melanoma and non-small-cell lung cancer patients who initially exhibited an objective response upon anti-PD-1 treatment [8,9]. The mechanisms of intrinsic resistance to ICBT are well addressed in previous review articles [7,10]. Here, we specifically discuss the most comprehensively described mechanisms of acquired resistance and identify the major challenges in understanding and overcoming acquired resistance to ICBT.

## 2. Mechanisms of Acquired ICBT Resistance

**Figure 1:**
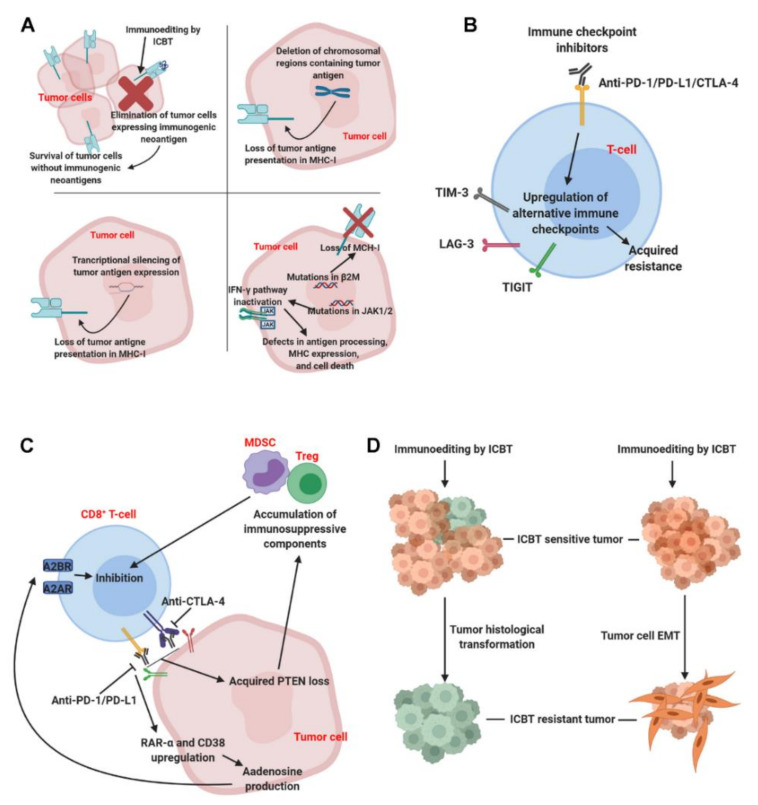
Overview of major mechanisms causing acquired resistance to immune checkpoint blockade therapy. Response to immune checkpoint blockade therapy (ICBT) is tightly controlled. The current literature has revealed several potential mechanisms contributing to acquired ICBT resistance. Panel (**A**), upper left: selective elimination of tumor cells with immunogenic neoantigens; upper right: loss of neoantigens due to chromosomal region deletion; lower left: loss of neoantigens due to transcriptional silencing; lower right: dysfunction of antigen processing and presentation. Panel (**B**): alternative immune checkpoints expression induced by ICBT. Panel (**C**): the coupling of tumor cell pathways stimulated by ICBT, such as adenosine production and PTEN loss, with the function of T cells. Panel (**D**): the transformation of tumor histological types and tumor cell epithelial–mesenchymal transition (EMT) after ICBT treatment. Abbreviations: MHC: major histocompatibility complex; PD-1: programmed cell death protein 1; PD-L1: programmed death-ligand 1; CTLA-4: cytotoxic T-lymphocyte-associated protein 4; TIM-3: T-cell immunoglobulin and mucin-domain containing-3; LAG-3: lymphocyte-activation gene 3; TIGIT: T-cell immunoreceptor with Ig and ITIM domains; MDSC: myeloid-derived suppressive cell; Treg: regulatory T-cell; A2AR: adenosine A2A receptor; A2BR: adenosine 2b receptor.

Increasing evidence suggests that the efficacy of ICBT is regulated by both tumor intrinsic factors and tumor extrinsic factors [11,12]. Mechanisms of acquired resistance to ICBT have been discovered through tumor tissue sequencing performed pre-and post-treatment, and currently, most evidence points to mutations in tumor cells that affect the IFNγ signaling pathways, antigen expression, and antigen presentation complexes. Investigations of tumor-infiltrating T cells have also revealed the upregulation of alternative immune checkpoint genes after anti-PD-1 treatment. Meanwhile, recent studies pointed out that coupling between tumor cells and T cells promoted the development of acquired resistance to ICBT (discussed below). Here, we discuss the validated mechanisms that are associated with acquired resistance to ICBT (Figure 1).

### 2.1. Loss of Tumor Cell Visibility to Immune Cells

Tumor cell recognition by the tumor-infiltrating T cells is an essential step in T-cell mediated tumor elimination [13]. For tumor-infiltrating T cells to recognize tumor cells, the tumor antigens must be properly processed and presented on major histocompatibility complex (MHC) molecules. Analyses of tumor tissues that developed acquired resistance to ICBT have shown that these tumor cells have defects in antigen presentation. These defects can be categorized into two groups: (i) Loss of immunogenic neoantigens and (ii) dysfunction of antigen processing and presentation.

Neoantigens are antigens encoded by mutated tumor-specific genes. One consensus regarding immunotherapy is that high mutational load is positively correlated with neoantigen levels and ICBT response in human tumors [14,15]. In a recent study, Mandal et al. artificially increased tumor clonal mutation loads in mouse cell lines by knocking out the mismatch repair genes and observed impressive antitumor immunity and response to ICBT. These data recapitulate the major findings in human studies and directly validate the theory that clonal mutational load (neoantigen sources) is a major driving force of antitumor immunity and immunotherapeutic efficacy [16]. However, the landscape of tumor neoantigens evolves during interactions between tumors and T cells. T-cell populations exert selective pressure on tumor cells that can sculpt the antigenicity of tumors, resulting in the emergence of tumor cells with minimal neoantigen expression [17,18]. Therefore, the strong therapeutic T-cell response induced by ICBT can cause sensitive tumors to lose neoantigens and eventually become invisible to the immune system.

A study of the evolving landscape of tumor neoantigens during ICBT confirmed that loss of neoantigens is a potential mechanism of acquired ICBT resistance. Genomic analyses were performed on patient-matched tumor tissues (i.e., before vs. after ICBT) from non-small-cell lung cancers. Tumors with acquired ICBT resistance showed genomic changes resulting in the loss of 7 to 18 validated, mutation-associated neoantigens [19]. Furthermore, the lost neoantigens demonstrated higher predicted MHC binding affinity and T-cell stimulating efficacy than neoantigens that were either retained or gained in resistant tumors [19]. In a study of advanced melanoma, anti-PD-1 treatment lowered the predicted mutational and neoantigen burden in treatment-sensitive tumors [20]. Similar observations were made in both mouse models treated with anti-PD-1 and in human tumors treated with adoptive T-cell transfer [16,18]. Mechanistically, the elimination of tumor subclones or deletion of chromosomal regions containing truncal alterations were identified as two potential causes of neoantigen loss [19]. A recent study used an artificial neoantigen, ovalbumin (OVA), to study the mechanisms of antigen expression silencing [21]. Mouse tumors having developed acquired resistance to adoptive cell therapy showed loss of OVA expression. Transcriptional silencing was identified as the major underlying mechanism of OVA antigen loss [21]. Administration of DNA methyltransferase inhibitors restored antigen expression [21], which suggests that combining these agents with immunotherapies improves long-term efficacy. This mechanism was primarily identified in a mouse model of adoptive cell therapy; however, it may also hold true for ICBT, considering that resistant mechanisms could be shared among different immunotherapies. Taken together, these data demonstrate that ICBT drives tumor mutation and neoantigen burden loss, which potentially lead to acquired drug resistance.

The immunogenicity and quantity of neoantigens present in tumor cells are the fundamental elements for cancer immunity. However, the antigen presentation process determines the direct interaction between tumor cells and tumor-specific T cells. In tumor cells, the IFN-γ signaling pathway is known to directly upregulate antigen-presenting machinery by upregulating proteasome subunits, transporters associated with antigen processing, and MHC-I molecules [22]. Tumor cells with an active IFN-γ signaling pathway can respond to IFN-γ secreted by immune cells in the tumor microenvironment, and, thus, become more visible to CD8^+^ T cells [23,24]. However, dysfunction in the IFN-γ signaling pathway has been identified in tumors that are resistant to ICBT [8]. Whole-exome sequencing of tumors with acquired resistance to anti-PD-1 treatment revealed loss-of-function mutations and wild-type deletion in Janus kinases 1 and 2 (JAK1/JAK2), which are activated upon IFN-γ binding to IFN receptors [8]. Loss of functional JAK1/JAK2 kinases impedes phosphorylation of STATs, transcription factors that control the expression of genes such as MHC class I and antigen peptide transporter 1 [22].

Another mutation involved in MHC-I expression was identified in tumors with acquired ICBT resistance. As a key component of the MHC-I complex, beta-2-microglobulin (β2M) expression is required for stabilizing the alpha-subunits of the MHC-I protein. In patients that developed acquired anti-PD-1 resistance, a truncating mutation discovered in the β2M gene leads to loss of surface expression of the MHC-I complex [8,25,26]. These data demonstrate that dysfunctional tumor antigen-presenting machinery after primary ICBT response reduces tumor cell visibility to immune cells, leading to acquired ICBT resistance.

### 2.2. Loss of Tumor Cells Sensitivity to Immune Effectors

Tumor sensitivity to immune effector cells is the ultimate determinant of immunotherapeutic success. IFN-γ is a cytokine with multiple functions in antitumor immunity. In addition to its effects on antigen presentation, IFN-γ also displays direct anticancer activity. Mechanistically, the IFN-γ signaling pathways upregulate both p21 and p27 molecules to arrest the cell cycle, and induce apoptotic cell death [27,28]. Mutations and loss of heterozygosity (LOH) in the IFN-γ signaling pathways in tumor cells quench an important immune-cell-mediated tumor cell death mechanism, thus potentially accelerating acquired resistance to ICBT.

It is also important to note the complexity of IFN-γ signaling in tumor immunity. This pathway affects expression of more than 200 genes, many of which are involved in cancer cell immune evasion, including PD-L1, PD-L2, and non-classical MHC class Ib antigens, indoleamine 2,3-dioxygenase (IDO), and many others [29,30]. Thus, inactivation of the IFN-γ signaling pathway after ICBT likely has more complicated effects on the antitumor immune network than simply reducing tumor antigen presentation and inhibiting tumor cell death. In summary, published studies indicate a correlation between IFN-γ signaling and acquired ICBT resistance; however, further studies are required to confirm that the loss of IFN-γ signaling causes such a resistance.

### 2.3. Compensation of Alternative Immune Checkpoints on T Cells

Many studies have demonstrated that alternative immune checkpoint genes, besides PD-1/PD-L1 and CTLA-4, are not only sufficient to induce T-cell dysfunction but are also highly expressed on T cells that acquire resistance to ICBT. Two fully immunocompetent, genetically engineered lung cancer mouse models were used to model acquired resistance to anti-PD-1 [31]. Notably, tumors with acquired anti-PD-1 resistance upregulated T-cell immunoglobulin and mucin domain 3 (TIM-3) on both CD4^+^ and CD8^+^ T cells. However, other immune-suppressive checkpoint genes—including lymphocyte-activation gene 3 (LAG-3) and CTLA-4—and immunosuppressive components—such as T-reg, tumor-associated alveolar macrophages, and tumor-associated neutrophils—were unchanged. Further analysis indicated that TIM-3 upregulation is specifically induced by anti-PD-1 treatment and high TIM-3 expression is found in anti-PD-1 antibody bonded T cells. Additionally, the upregulation of TIM-3 was observed in two patients with resistance after anti-PD-1 treatment. In tumor models, the administration of TIM-3 blockades provided additional clinical benefit in PD-1-resistant tumors [31]. Similarly, compensatory expression of T-cell immunoreceptors with Ig and ITIM domains (TIGIT) on tumor antigen-specific CD8^+^ T cells was induced by anti-PD-1 treatment in melanoma patients [32]. Interestingly, a study in classical Hodgkin lymphoma revealed that PD-1 is overexpressed in the tumor microenvironment after anti-PD-1 acquired resistance [33].

Collectively, these studies showed that ICBT potentially induces compensation of both alternative and targeted immune checkpoint genes, leading to acquired resistance. Notably, TIM-3 upregulation was observed specifically in tumors relapsing after anti-PD-1 treatment but not with anti-CTLA-4 treatment [31]. These observations suggest that the compensation may not be a universal mechanism of acquired resistance and different immune checkpoint genes can be specifically stimulated by different ICBT. However, the detailed molecular signaling pathways leading to compensatory immune checkpoint upregulation in acquired ICBT resistance are not yet well understood. Understanding these aspects will clarify whether specific ICBT induces particular alternative immune checkpoint expression patterns and provide a rationale for designing combination treatments. Other alternative immune checkpoints, such as LAG-3 and IDO, were associated with intrinsic ICBT resistance [34,35,36,37]. However, their roles in acquired ICBT resistance were unconfirmed.

### 2.4. Tumor Cell-Mediated T-Cell Suppression

The interactions between activated T cells and tumor cells have been highlighted in the development of acquired resistance to ICBT. Chen and colleagues identified the mechanism by which CD38 expression was upregulated on tumors treated by PD-1/PD-L1 blockades [38]. Blocking PD-1/PD-L1 increased all-trans retinoic acid (ATRA) and type I IFN in the treatment-sensitive TME, which leads to activation of the retinoic acid receptor alpha (RAR-α) and subsequently CD38 expression on tumor cells. CD38 on tumor cells then converts tumor microenvironmental NAD^+^ to immunosuppressive adenosine via the CD38/CD203a/CD73 pathway. Consequently, the adenosine produced by tumor cells activates A2A and A2B adenosine receptors on CD8^+^ T cells to suppress their antitumor functions [38]. Chen and colleagues tested whether blocking either CD38 or the adenosine receptor could improve the therapeutic outcome of anti-PD-L1 in mouse models of cancer. Co-inhibition of either CD38 or the A2A/A2B adenosine receptors with PD-L1 showed superior antitumor efficacy over monotherapy alone [38]. These findings defined a feedback loop between tumor-infiltrating T cells and tumor cells in the context of primary response and subsequent resistance to ICBT. However, the current studies did not investigate other catalytic pathways that also generate adenosine. How these different adenosine-generating pathways are regulated in tumors displaying acquired anti-PD-1/PD-L1 resistance also requires further investigation.

Beyond the adenosine pathway, CD80 expression on tumor stem cells responsive to transforming growth factor-β (TGF-β) was identified as the primary cause of relapse after adoptive T-cell transfer [39]. In a skin cancer model for squamous cell carcinoma that responds to adoptive T-cell transfer, TGF-β-responding tumor stem cells selectively expressed CD80, a surface ligand previously identified on immune cells. CD80 expression on tumor stem cells directly decreases T-cell activity upon engaging with CTLA-4 [39]. On the other hand, high levels of CD80 can trap PD-L1, thus alleviating its suppressive effects on PD-1 expressing T cells [40]. This suggests that one molecule may have opposing impacts on T-cell function in the complex co-stimulation network. Based on the current evidence, we can postulate that, with PD-L1 blockade, CD80 may exert immunosuppressive effects on T cells by binding to CTLA-4, thereby leading to acquired anti-PD-L1 resistance. However, whether such a putative mechanism exists in the ICBT resistant scenario needs further validation.

Accumulating evidence shows that oncogenic pathways can influence the cross-talk between cancer cells and the surrounding tumor microenvironment [41]. Loss of the PTEN tumor suppressor gene is commonly observed in numerous types of cancers and is considered as a mechanism causing intrinsic resistance to ICBT. The tumor microenvironment of PTEN-deficient tumors shows an increased percentage of regulatory T cells and a concurrent decrease in cytotoxic T-cell frequency [42,43,44]. In a patient with metastatic melanoma who initially showed a durable partial response to combined anti-CTLA-4 + anti-PD-1 therapy but subsequently developed resistance, acquired PTEN loss was detected in the drug-resistant tumors [45]. T-cell exclusion was observed in the acquired resistant tumor [45]. These data confirmed that acquired resistance to ICBT can arise upon the selection/induction of oncogenic pathway mutations in tumor cells that mediate immunosuppression. Other oncogenic pathways that have contributed to intrinsic resistance [41] might also contribute to acquired resistance, and evaluation for those mutations should be considered at the time of relapse in patients developing acquired resistance.

### 2.5. Tumor Pathologic Transformation and Epithelial–Mesenchymal Transition

Transformation of tumor pathological subtypes is a phenomenon documented during cancer treatment. It was known that lung adenocarcinoma can be transformed into neuroendocrine carcinoma under epidermal growth factor receptor (EGFR)-tyrosine kinase inhibitor (TKI) treatment, leading to resistance in EGFR-mutated non-small-cell lung cancers [46,47]. Similarly, non-small-cell lung cancer that developed acquired ICBT resistance demonstrated transformation to small cell lung cancer [48,49]. These non-small-cell lung cancers showed neuroendocrine features before ICBT [48], suggesting that immunoediting induced by ICBT selectively eliminated the treatment-sensitive tumor cells and can be a reason for the histological transformation. However, due to the limited sample size [48,49], we cannot yet conclude the prevalence of this mechanism.

In addition to tumor histological transformation, the epithelial–mesenchymal transition (EMT) in individual cancer cells may be another cause of acquired ICBT resistance. EMT is a process by which epithelial tumor cells gain the morphology and properties of mesenchymal stem cells. During the EMT, epithelial cells lose their cell polarity and cell–cell adhesion and acquire migratory and invasive capabilities [50]. In melanoma models, tumors undergoing EMT lack E-cadherin expression and thus are resistant to anti-PD-1 and anti-CTLA-4 therapies [51]. Mechanistic studies showed that E-cadherin is the primary ligand of CD103, which is expressed on resident memory T cells, dendritic cells, and other immune cells [51,52,53,54]. Loss of E-cadherin expression will inevitably impair the CD103^+^ immune-cell-mediated immune response. Based on these data, we speculate that the EMT process can shift treatment responsive tumors to become resistant, resulting in acquired ICBT resistance.

## 3. Detection of Acquired ICBT Resistance

Although acquired ICBT resistance is well known to clinicians, its detection and prediction are still challenging. Currently, the Response Evaluation Criteria in Solid Tumors (RECIST 1.1) is routinely used to evaluate response to cytotoxic cancer therapies by radiographic imaging. However, the atypical response patterns seen in some cases undergoing immunotherapy limit the accuracy of RECIST 1.1 in evaluating cancer immunotherapy response. Since RECIST 1.1 generally classifies significant tumor growth and/or newly detectable tumor lesions as a progressive disease, it fails to recognize the potential pseudoprogression during immunotherapy and long-term effectiveness of immunotherapy. For this reason, an immune-related response criteria termed iRECIST was developed in 2017 to assess the growth patterns unique to patients treated with immunotherapy [55]. The basic definition of measurable or non-measurable tumor lesions and assessing tumor responses remains the same in iRECIST as in RECIST 1.1. The major change in iRECIST is the introduction of ‘unconfirmed progressive disease’ to describe an initial increase in tumor size or the emergence of new lesions. An additional follow-up is needed to confirm or withdraw an ‘unconfirmed progressive disease’ designation. These new criteria will diminish erroneous terminations of immunotherapies and unjustified patient exclusion from clinical studies due to tumor pseudoprogression. However, neither of these radiographic assessment-based criteria can provide early diagnosis of acquired resistance nor provide any mechanistic insights. Novel detection methods that identify potential acquired resistance before radiographic tumor progression would significantly impact patient care.

Recent improvements in the understanding of acquired resistance mechanisms make novel resistance detection before radiographic tumor progression increasingly achievable. To this end, histologic analysis and immune profiling analyses by next-generation sequencing and high-throughput flow cytometry/mass cytometry of tumors were developed to serve as a robust predictor of acquired resistance to ICBT [56,57]. By performing serial biopsies after ICBT treatment, these novel methods make it possible to monitor tumor immunoediting post-ICBT treatment. The changes in PD-L1 expression, cell populations, immune gene expressions, and the tumor microenvironment may predict acquired resistance and case-specific mechanisms.

Less invasive methods are also being studied to monitor tumor responses to ICBT, including testing for circulating tumor DNA (ctDNA) and T-cell receptor (TCR) sequencing on circulating T cells. In cancer patients, ctDNA amounts have been correlated with tumor burden after ICBT [58,59,60,61]. Furthermore, tumor-specific mutations can be detected from the ctDNA of cancer patients [62]. These features of ctDNA form a theoretical foundation upon which to apply ctDNA testing to monitor ICBT efficacy. The changes in ctDNA precede radiographic evidence of responses to ICBT [58]. TCR sequencing can provide a more direct evaluation of the tumor-specific T-cell populations. The TCR repertoire and its features are associated with survival in immunotherapy-treated cancers [63,64].

Molecular imaging by nuclear medicine equipment provides another non-invasive method to measure immune protein expression on tumor tissues. Recent pre-clinical and early-stage clinical studies showed the feasibility of using isotope-labeled PD-1- and PD-L1-targeted imaging agents for quantitative, real-time assessment of PD-1/PD-L1 expression in the tumor environment [65,66]. However, whole-tumor molecular imaging cannot discriminate the tumor and immune cells in the tumor tissue. Since immune checkpoints have distinct functions on different cell types, results of whole-tumor molecular imaging may not provide an accurate evaluation of anti-tumor immune mechanisms. Despite such caveats, molecular imaging still shows huge potential for measuring immune-protein-related biomarkers in patients who are not suitable for biopsies [66]. Several clinical trials (e.g., ClinicalTrials.gov identifiers: NCT04222426 and NCT04006522) are ongoing to determine the value of immune-protein-specific molecular imaging in predicting ICBT response.

These novel cellular and molecular tests may eventually lead to earlier diagnoses of acquired ICBT resistance than the standard RECIST 1.1/iRECIST; however, they are not currently performed in routine clinical practice (Table 1). The reproducibility of these novel measurements has yet to be studied systematically, and further randomized clinical trials are necessary to validate their predictive value on acquired ICBT resistance in multiple cohorts.

## 4. Principles for Overcoming Acquired ICBT Resistance

The development of acquired ICBT resistance in patients is usually limited to one or few sites of disease [8,9,77]. One recent observational study found that, among all metastatic tumor sites, the lymph nodes are the most common site of acquired resistance in patients with advanced non-small-cell lung cancer [77]. Therapeutic regimens administered for progressive disease secondary to acquired ICBT resistance largely depend on tumor burden, location, and physician experience. Therapies include both systemic (first/second-line chemotherapy and targeted therapy) and local treatments as well as re-challenging with the original ICBT agent [77]. In this study, these second-line therapies were able to delay tumor progression [77]; however, further clinical trials are needed to establish standardized treatment regimens for patients with acquired ICBT resistance.

With improved understanding of the underlying mechanisms leading to acquired ICBT resistance, efforts are being made to derive individualized strategies to combat this resistance. One major focus is to target the compensatory mechanisms, such as TIM-3 and the adenosine metabolic/signaling pathways, that cause T-cell exhaustion upon anti-PD-1/PD-L1 or anti-CTLA-4 treatments. Data from pre-clinical models has demonstrated the efficacy of such combination therapies [31,38]. However, phase II/III clinical trial data which support the combination of alternative immune checkpoint inhibitors to overcome the acquired anti-PD-1/PD-L1/CTLA-4 resistance have yet to be reported. Additionally, the possible activation of specific oncogenic pathways in tumors that have acquired ICBT resistance [45] raises the prospect for administering inhibitors of these pathways to restore T-cell infiltration and cancer cell depletion. Transcriptional regulatory drugs, such as azacytidine and decitabine, might be effective in tumors with transcriptional silencing of tumor antigen expression [21]. Despite the many theoretic possibilities, the mechanisms that drive acquired ICBT resistance in individual patients are usually unknown, making it difficult to develop a precise and individualized combination therapy in current clinical practice.

## 5. Conclusions and Future Directions

The past decade has witnessed the revolutionary success of ICBT in selected cancer types. However, intrinsic resistance to ICBT was observed in the majority of cancer patients, significantly limiting the number of beneficiaries. Other patients showed an initial response but eventually acquired resistance to ICBT, which ultimately diminished their long-term survival. Clinical investigations of cancer relapse following ICBT have begun to identify the key mechanisms of acquired resistance from the studies of tumor cells, immune cells, and their interactions. Key challenges remain in fully understanding and efficiently treating acquired resistance.

One poorly understood fundamental question is how acquired ICBT resistance is initiated in individual patients. One possibility is that ICBT selectively kills only intrinsically sensitive tumor cells leaving resistant cell populations to rebound, resulting in clinical relapse. The main driver for this type of acquired resistance is the small proportion of tumor cells that are resistant to ICBT from the onset of treatment. This hypothesis is supported by two recent publications showing that the highly heterogeneous nature of both tumor cell populations and tumor microenvironmental components creates different compartments within the tumor tissue, each with diverse sensitivities to T-cell killing [78,79]. Resistance due to this mechanism may be avoided by designing combination therapies which eliminate multiple tumor cell subclones from the start of treatment. Another potential pathway causing resistance to ICBT is acquired by tumor cells during the treatment. The accumulation of mutations in tumor cells, tumor-immune cell interactions, and reshaping of the tumor microenvironment during ICBT may transform sensitive tumors into resistant tumors. Differentiating between these two initiation pathways may allow for the development and implementation of specific strategies to avoid acquired ICBT resistance.

Another critical question is whether intermittent ICBT could sustain long term responses and help avoid acquired resistance. The induction of compensatory immunosuppressive mechanisms after anti-PD-1/PD-L1 and anti-CTLA-4 suggests that sustained ICBT may induce feedback inhibition [26,31,38]. It seems that these compensatory immunosuppressive factors are mainly controlled by epigenetic mechanisms, which are highly reversible. However, it is still unknown if these compensatory immunosuppressive mechanisms will subside after discontinuation of ICBT. Clinical data from non-small-cell lung cancer patients demonstrated that some relapsed tumors could respond to the original ICBT [77], indicating that tumor cells with acquired resistance can regain sensitivity after a certain period of time off treatment. If this hypothesis is true, intermittent ICBT may be a better option than sustained therapy, providing both reduced toxicity and improved long-term tumor control.

Extensive studies have revealed key mechanisms causing tumor intrinsic resistance to ICBT. However, whether these same mechanisms can be activated during ICBT and lead to acquired resistance is largely unknown. For example, dysbiosis to the gut microbiome can impair immune functions and thus affect ICBT response [80,81,82]. The impacts of the microbiome on intrinsic ICBT resistance have been widely accepted, but whether the patients’ microbiome can shift from an ‘ICBT sensitive pattern’ to an ‘ICBT resistant pattern’ during treatment is unclear. Recent studies also demonstrated that exosome-derived PD-L1 avoids PD-1 axis inhibition, thus giving rise to intrinsic resistance [83,84]. It is important to know if the exosome packaged PD-L1 is upregulated in the development of acquired resistance to ICBT. Considering the universalism of tumor–immune cell interactions, the same mechanism can likely induce both intrinsic and acquired resistance. Validating the key mechanisms of intrinsic ICBT resistance in acquired resistance will significantly accelerate our understanding of acquired ICBT resistance.

Finally, the objective difficulties of investigating acquired ICBT resistance must be properly addressed. One major issue is tissue acquisition of tumors with acquired resistance. To study intrinsic resistance, primary tumor tissues that are resected during surgical treatment are valuable sources for mechanistic studies. Most animal tumor models are also suitable for studying intrinsic resistance. However, these sources do not necessarily exist for acquired resistance studies, which require paired tumor tissues from pre- and post-treatment. Biopsies of recurrent tumor tissues are sufficient for diagnosis but not for comprehensive investigation utilizing multiple techniques such as high-throughput sequencing, immunostaining, and flow cytometry. This restriction has significantly limited the depth and scope of current acquired ICBT resistance studies [33,45]. Addressing these challenges through more coordinated and robust tissue collection efforts or the development of novel mouse models that represent acquired ICBT resistance will provide additional opportunities to understand and ultimately defeat relapsed cancers.

## Figures and Tables

**Table 1:** Strategies under development for evaluation of immune checkpoint blockade therapy.

Approaches	Mechanisms of Approach	Relevance to Anti-Tumor Immune Mechanisms	Reference
Radiographic imaging	Direct measurement of tumor burden	No	[55]
Molecular imaging	Measurement of immune protein expression on whole tumor tissues	Yes	[65,66]
Serum tumor biomarkers	Estimation of tumor burden by quantifying serum tumor antigens or genetic material	Marker-dependent	[67,68]
Circulating tumor cells	Estimation of tumor burden by quantifying and featuring circulating tumor cells	Marker-dependent	[69]
PD-L1 expression	Assessment of the proportion of PD-L1-positive tumor cells, immune cells, or both on tumor tissue sections	Yes	[70,71]
Tumor-infiltrating lymphocyte	Assessment of T cells at invasive tumor margin or tumor parenchyma	Yes	[72,73]
T-cell receptor sequencing	Assessment of T-cell clonality by sequencing of T-cell receptor β-chain	Yes	[74]
Mutational and neoantigen burden	Exome sequencing to assess non-synonymous somatic mutations with antigenic prediction	Yes	[15]
DNA mismatch repair genes status	Assessment of mismatch repair genes status in tumor parenchyma	Yes	[75]
Immune gene signatures	Assessment of immune gene expression signature from the tumor microenvironment	Yes	[76]

## References

[1] Busch W. (1868). Aus der Sitzung der medicinischen Section vom 13 November 1867. Berl. Kli. Wochenschr..

[2] Coley W.B. (1991). The treatment of malignant tumors by repeated inoculations of erysipelas. With a report of ten original cases. 1893. Clin. Orthop. Relat. Res..

[3] Mellman I., Coukos G., Dranoff G. (2011). Cancer immunotherapy comes of age. Nature.

[4] Palucka A.K., Coussens L.M. (2016). The Basis of Oncoimmunology. Cell.

[5] Wei S.C., Duffy C.R., Allison J.P. (2018). Fundamental Mechanisms of Immune Checkpoint Blockade Therapy. Cancer Discov..

[6] Le D.T., Uram J.N., Wang H., Bartlett B.R., Kemberling H., Eyring A.D., Skora A.D., Luber B.S., Azad N.S., Laheru D. (2015). PD-1 Blockade in Tumors with Mismatch-Repair Deficiency. N. Engl. J. Med..

[7] Zhao X., Subramanian S. (2017). Intrinsic Resistance of Solid Tumors to Immune Checkpoint Blockade Therapy. Cancer Res..

[8] Zaretsky J.M., Garcia-Diaz A., Shin D.S., Escuin-Ordinas H., Hugo W., Hu-Lieskovan S., Torrejon D.Y., Abril-Rodriguez G., Sandoval S., Barthly L. (2016). Mutations Associated with Acquired Resistance to PD-1 Blockade in Melanoma. N. Engl. J. Med..

[9] Horn L., Spigel D.R., Vokes E.E., Holgado E., Ready N., Steins M., Poddubskaya E., Borghaei H., Felip E., Paz-Ares L. (2017). Nivolumab Versus Docetaxel in Previously Treated Patients With Advanced Non-Small-Cell Lung Cancer: Two-Year Outcomes From Two Randomized, Open-Label, Phase III Trials (CheckMate 017 and CheckMate 057). J. Clin. Oncol..

[10] Kalbasi A., Ribas A. (2020). Tumour-intrinsic resistance to immune checkpoint blockade. Nat. Rev. Immunol..

[11] Sharma P., Hu-Lieskovan S., Wargo J.A., Ribas A. (2017). Primary, Adaptive, and Acquired Resistance to Cancer Immunotherapy. Cell.

[12] Syn N.L., Teng M.W.L., Mok T.S.K., Soo R.A. (2017). De-novo and acquired resistance to immune checkpoint targeting. Lancet Oncol..

[13] Chen D.S., Mellman I. (2013). Oncology meets immunology: the cancer-immunity cycle. Immunity.

[14] Chan T.A., Yarchoan M., Jaffee E., Swanton C., Quezada S.A., Stenzinger A., Peters S. (2019). Development of tumor mutation burden as an immunotherapy biomarker: utility for the oncology clinic. Ann. Oncol..

[15] McGranahan N., Furness A.J., Rosenthal R., Ramskov S., Lyngaa R., Saini S.K., Jamal-Hanjani M., Wilson G.A., Birkbak N.J., Hiley C.T. (2016). Clonal neoantigens elicit T cell immunoreactivity and sensitivity to immune checkpoint blockade. Science.

[16] Mandal R., Samstein R.M., Lee K.W., Havel J.J., Wang H., Krishna C., Sabio E.Y., Makarov V., Kuo F., Blecua P. (2019). Genetic diversity of tumors with mismatch repair deficiency influences anti-PD-1 immunotherapy response. Science.

[17] Rosenthal R., Cadieux E.L., Salgado R., Bakir M.A., Moore D.A., Hiley C.T., Lund T., Tanic M., Reading J.L., Joshi K. (2019). Neoantigen-directed immune escape in lung cancer evolution. Nature.

[18] Verdegaal E.M., de Miranda N.F., Visser M., Harryvan T., van Buuren M.M., Andersen R.S., Hadrup S.R., van der Minne C.E., Schotte R., Spits H. (2016). Neoantigen landscape dynamics during human melanoma-T cell interactions. Nature.

[19] Anagnostou V., Smith K.N., Forde P.M., Niknafs N., Bhattacharya R., White J., Zhang T., Adleff V., Phallen J., Wali N. (2017). Evolution of Neoantigen Landscape during Immune Checkpoint Blockade in Non-Small Cell Lung Cancer. Cancer Discov..

[20] Riaz N., Havel J.J., Makarov V., Desrichard A., Urba W.J., Sims J.S., Hodi F.S., Martin-Algarra S., Mandal R., Sharfman W.H. (2017). Tumor and Microenvironment Evolution during Immunotherapy with Nivolumab. Cell.

[21] Wylie B., Chee J., Forbes C.A., Booth M., Stone S.R., Buzzai A., Abad A., Foley B., Cruickshank M.N., Waithman J. (2019). Acquired resistance during adoptive cell therapy by transcriptional silencing of immunogenic antigens. Oncoimmunology.

[22] Zhou F. (2009). Molecular mechanisms of IFN-gamma to up-regulate MHC class I antigen processing and presentation. Int. Rev. Immunol..

[23] Martini M., Testi M.G., Pasetto M., Picchio M.C., Innamorati G., Mazzocco M., Ugel S., Cingarlini S., Bronte V., Zanovello P. (2010). IFN-gamma-mediated upmodulation of MHC class I expression activates tumor-specific immune response in a mouse model of prostate cancer. Vaccine.

[24] Propper D.J., Chao D., Braybrooke J.P., Bahl P., Thavasu P., Balkwill F., Turley H., Dobbs N., Gatter K., Talbot D.C. (2003). Low-dose IFN-gamma induces tumor MHC expression in metastatic malignant melanoma. Clin. Cancer Res..

[25] Sade-Feldman M., Jiao Y.J., Chen J.H., Rooney M.S., Barzily-Rokni M., Eliane J.P., Bjorgaard S.L., Hammond M.R., Vitzthum H., Blackmon S.M. (2017). Resistance to checkpoint blockade therapy through inactivation of antigen presentation. Nat. Commun..

[26] Gettinger S., Choi J., Hastings K., Truini A., Datar I., Sowell R., Wurtz A., Dong W., Cai G., Melnick M.A. (2017). Impaired HLA Class I Antigen Processing and Presentation as a Mechanism of Acquired Resistance to Immune Checkpoint Inhibitors in Lung Cancer. Cancer Discov..

[27] Hobeika A.C., Etienne W., Torres B.A., Johnson H.M., Subramaniam P.S. (1999). IFN-gamma induction of p21(WAF1) is required for cell cycle inhibition and suppression of apoptosis. J. Interferon Cytokine Res..

[28] Harvat B.L., Seth P., Jetten A.M. (1997). The role of p27Kip1 in gamma interferon-mediated growth arrest of mammary epithelial cells and related defects in mammary carcinoma cells. Oncogene.

[29] Mojic M., Takeda K., Hayakawa Y. (2017). The Dark Side of IFN-gamma: Its Role in Promoting Cancer Immunoevasion. Int. J. Mol. Sci..

[30] Abiko K., Matsumura N., Hamanishi J., Horikawa N., Murakami R., Yamaguchi K., Yoshioka Y., Baba T., Konishi I., Mandai M. (2015). IFN-gamma from lymphocytes induces PD-L1 expression and promotes progression of ovarian cancer. Br. J. Cancer.

[31] Koyama S., Akbay E.A., Li Y.Y., Herter-Sprie G.S., Buczkowski K.A., Richards W.G., Gandhi L., Redig A.J., Rodig S.J., Asahina H. (2016). Adaptive resistance to therapeutic PD-1 blockade is associated with upregulation of alternative immune checkpoints. Nat. Commun..

[32] Chauvin J.M., Pagliano O., Fourcade J., Sun Z., Wang H., Sander C., Kirkwood J.M., Chen T.H., Maurer M., Korman A.J. (2015). TIGIT and PD-1 impair tumor antigen-specific CD8(+) T cells in melanoma patients. J. Clin. Invest..

[33] Sasse S., Reddemann K., Diepstra A., Oschlies I., Schnitter A., Borchmann S., Engert A., Borchmann P., Klapper W. (2019). Programmed cell death protein-1 (PD-1)-expression in the microenvironment of classical Hodgkin lymphoma at relapse during anti-PD-1-treatment. Haematologica.

[34] Woo S.R., Turnis M.E., Goldberg M.V., Bankoti J., Selby M., Nirschl C.J., Bettini M.L., Gravano D.M., Vogel P., Liu C.L. (2012). Immune inhibitory molecules LAG-3 and PD-1 synergistically regulate T-cell function to promote tumoral immune escape. Cancer Res..

[35] Bottai G., Raschioni C., Losurdo A., Di Tommaso L., Tinterri C., Torrisi R., Reis-Filho J.S., Roncalli M., Sotiriou C., Santoro A. (2016). An immune stratification reveals a subset of PD-1/LAG-3 double-positive triple-negative breast cancers. Breast Cancer Res..

[36] Chinn Z., Stoler M.H., Mills A.M. (2019). PD-L1 and IDO expression in cervical and vulvar invasive and intraepithelial squamous neoplasias: implications for combination immunotherapy. Histopathology.

[37] Mills A., Zadeh S., Sloan E., Chinn Z., Modesitt S.C., Ring K.L. (2018). Indoleamine 2,3-dioxygenase in endometrial cancer: a targetable mechanism of immune resistance in mismatch repair-deficient and intact endometrial carcinomas. Mod. Pathol..

[38] Chen L., Diao L., Yang Y., Yi X., Rodriguez B.L., Li Y., Villalobos P.A., Cascone T., Liu X., Tan L. (2018). CD38-Mediated Immunosuppression as a Mechanism of Tumor Cell Escape from PD-1/PD-L1 Blockade. Cancer Discov.

[39] Miao Y., Yang H., Levorse J., Yuan S., Polak L., Sribour M., Singh B., Rosenblum M.D., Fuchs E. (2019). Adaptive Immune Resistance Emerges from Tumor-Initiating Stem Cells. Cell.

[40] Sugiura D., Maruhashi T., Okazaki I.M., Shimizu K., Maeda T.K., Takemoto T., Okazaki T. (2019). Restriction of PD-1 function by cis-PD-L1/CD80 interactions is required for optimal T cell responses. Science.

[41] Zhao X., Subramanian S. (2018). Oncogenic pathways that affect antitumor immune response and immune checkpoint blockade therapy. Pharmacol. Ther..

[42] Zhao J., Chen A.X., Gartrell R.D., Silverman A.M., Aparicio L., Chu T., Bordbar D., Shan D., Samanamud J., Mahajan A. (2019). Immune and genomic correlates of response to anti-PD-1 immunotherapy in glioblastoma. Nat. Med..

[43] Vidotto T., Saggioro F.P., Jamaspishvili T., Chesca D.L., Picanco de Albuquerque C.G., Reis R.B., Graham C.H., Berman D.M., Siemens D.R., Squire J.A. (2019). PTEN-deficient prostate cancer is associated with an immunosuppressive tumor microenvironment mediated by increased expression of IDO1 and infiltrating FoxP3+ T regulatory cells. Prostate.

[44] Piro G., Carbone C., Carbognin L., Pilotto S., Ciccarese C., Iacovelli R., Milella M., Bria E., Tortora G. (2019). Revising PTEN in the Era of Immunotherapy: New Perspectives for an Old Story. Cancers.

[45] Trujillo J.A., Luke J.J., Zha Y., Segal J.P., Ritterhouse L.L., Spranger S., Matijevich K., Gajewski T.F. (2019). Secondary resistance to immunotherapy associated with beta-catenin pathway activation or PTEN loss in metastatic melanoma. J. Immunother. Cancer.

[46] Oser M.G., Niederst M.J., Sequist L.V., Engelman J.A. (2015). Transformation from non-small-cell lung cancer to small-cell lung cancer: Molecular drivers and cells of origin. Lancet Oncol..

[47] Sequist L.V., Waltman B.A., Dias-Santagata D., Digumarthy S., Turke A.B., Fidias P., Bergethon K., Shaw A.T., Gettinger S., Cosper A.K. (2011). Genotypic and histological evolution of lung cancers acquiring resistance to EGFR inhibitors. Sci. Transl. Med..

[48] Bar J., Ofek E., Barshack I., Gottfried T., Zadok O., Kamer I., Urban D., Perelman M., Onn A. (2019). Transformation to small cell lung cancer as a mechanism of resistance to immunotherapy in non-small cell lung cancer. Lung Cancer.

[49] Imakita T., Fujita K., Kanai O., Terashima T., Mio T. (2017). Small cell lung cancer transformation during immunotherapy with nivolumab: A case report. Respir. Med. Case Rep..

[50] Kalluri R., Weinberg R.A. (2009). The basics of epithelial-mesenchymal transition. J. Clin. Invest..

[51] Shields B.D., Koss B., Taylor E.M., Storey A.J., West K.L., Byrum S.D., Mackintosh S.G., Edmondson R., Mahmoud F., Shalin S.C. (2019). Loss of E-Cadherin Inhibits CD103 Antitumor Activity and Reduces Checkpoint Blockade Responsiveness in Melanoma. Cancer Res..

[52] Le Floc’h A., Jalil A., Vergnon I., Le Maux Chansac B., Lazar V., Bismuth G., Chouaib S., Mami-Chouaib F. (2007). Alpha E beta 7 integrin interaction with E-cadherin promotes antitumor CTL activity by triggering lytic granule polarization and exocytosis. J. Exp. Med..

[53] Nizard M., Roussel H., Diniz M.O., Karaki S., Tran T., Voron T., Dransart E., Sandoval F., Riquet M., Rance B. (2017). Induction of resident memory T cells enhances the efficacy of cancer vaccine. Nat. Commun..

[54] Roberts E.W., Broz M.L., Binnewies M., Headley M.B., Nelson A.E., Wolf D.M., Kaisho T., Bogunovic D., Bhardwaj N., Krummel M.F. (2016). Critical Role for CD103(+)/CD141(+) Dendritic Cells Bearing CCR7 for Tumor Antigen Trafficking and Priming of T Cell Immunity in Melanoma. Cancer Cell.

[55] Seymour L., Bogaerts J., Perrone A., Ford R., Schwartz L.H., Mandrekar S., Lin N.U., Litiere S., Dancey J., Chen A. (2017). iRECIST: guidelines for response criteria for use in trials testing immunotherapeutics. Lancet Oncol..

[56] Auslander N., Zhang G., Lee J.S., Frederick D.T., Miao B., Moll T., Tian T., Wei Z., Madan S., Sullivan R.J. (2018). Robust prediction of response to immune checkpoint blockade therapy in metastatic melanoma. Nat. Med..

[57] Jiang P., Gu S., Pan D., Fu J., Sahu A., Hu X., Li Z., Traugh N., Bu X., Li B. (2018). Signatures of T cell dysfunction and exclusion predict cancer immunotherapy response. Nat. Med..

[58] Goldberg S.B., Narayan A., Kole A.J., Decker R.H., Teysir J., Carriero N.J., Lee A., Nemati R., Nath S.K., Mane S.M. (2018). Early Assessment of Lung Cancer Immunotherapy Response via Circulating Tumor DNA. Clin. Cancer Res..

[59] Lee J.H., Long G.V., Boyd S., Lo S., Menzies A.M., Tembe V., Guminski A., Jakrot V., Scolyer R.A., Mann G.J. (2017). Circulating tumour DNA predicts response to anti-PD1 antibodies in metastatic melanoma. Ann. Oncol..

[60] Cabel L., Riva F., Servois V., Livartowski A., Daniel C., Rampanou A., Lantz O., Romano E., Milder M., Buecher B. (2017). Circulating tumor DNA changes for early monitoring of anti-PD1 immunotherapy: a proof-of-concept study. Ann. Oncol..

[61] Lipson E.J., Velculescu V.E., Pritchard T.S., Sausen M., Pardoll D.M., Topalian S.L., Diaz L.A. (2014). Circulating tumor DNA analysis as a real-time method for monitoring tumor burden in melanoma patients undergoing treatment with immune checkpoint blockade. J. Immunother. Cancer.

[62] Gandara D.R., Paul S.M., Kowanetz M., Schleifman E., Zou W., Li Y., Rittmeyer A., Fehrenbacher L., Otto G., Malboeuf C. (2018). Blood-based tumor mutational burden as a predictor of clinical benefit in non-small-cell lung cancer patients treated with atezolizumab. Nat. Med..

[63] Hopkins A.C., Yarchoan M., Durham J.N., Yusko E.C., Rytlewski J.A., Robins H.S., Laheru D.A., Le D.T., Lutz E.R., Jaffee E.M. (2018). T cell receptor repertoire features associated with survival in immunotherapy-treated pancreatic ductal adenocarcinoma. JCI Insight.

[64] Hogan S.A., Courtier A., Cheng P.F., Jaberg-Bentele N.F., Goldinger S.M., Manuel M., Perez S., Plantier N., Mouret J.F., Nguyen-Kim T.D.L. (2019). Peripheral Blood TCR Repertoire Profiling May Facilitate Patient Stratification for Immunotherapy against Melanoma. Cancer Immunol. Res..

[65] Broos K., Lecocq Q., Raes G., Devoogdt N., Keyaerts M., Breckpot K. (2018). Noninvasive imaging of the PD-1:PD-L1 immune checkpoint: Embracing nuclear medicine for the benefit of personalized immunotherapy. Theranostics.

[66] Bensch F., van der Veen E.L., Lub-de Hooge M.N., Jorritsma-Smit A., Boellaard R., Kok I.C., Oosting S.F., Schroder C.P., Hiltermann T.J.N., van der Wekken A.J. (2018). (89)Zr-atezolizumab imaging as a non-invasive approach to assess clinical response to PD-L1 blockade in cancer. Nat. Med..

[67] Sanmamed M., Perez-Gracia J., Schalper K., Fusco J., Gonzalez A., Rodriguez-Ruiz M., Onate C., Perez G., Alfaro C., Martín-Algarra S. (2017). Changes in serum interleukin-8 (IL-8) levels reflect and predict response to anti-PD-1 treatment in melanoma and non-small-cell lung cancer patients. Ann. Oncol..

[68] Diem S., Kasenda B., Spain L., Martin-Liberal J., Marconcini R., Gore M., Larkin J. (2016). Serum lactate dehydrogenase as an early marker for outcome in patients treated with anti-PD-1 therapy in metastatic melanoma. Br. J. Cancer.

[69] Tamminga M., de Wit S., Hiltermann T.J.N., Timens W., Schuuring E., Terstappen L.W., Groen H.J. (2019). Circulating tumor cells in advanced non-small cell lung cancer patients are associated with worse tumor response to checkpoint inhibitors. J. Immunother. Cancer.

[70] Borghaei H., Paz-Ares L., Horn L., Spigel D.R., Steins M., Ready N.E., Chow L.Q., Vokes E.E., Felip E., Holgado E. (2015). Nivolumab versus docetaxel in advanced nonsquamous non–small-cell lung cancer. N. Engl. J. Med..

[71] Garon E.B., Rizvi N.A., Hui R., Leighl N., Balmanoukian A.S., Eder J.P., Patnaik A., Aggarwal C., Gubens M., Horn L. (2015). Pembrolizumab for the treatment of non–small-cell lung cancer. N. Engl. J. Med..

[72] Chen P.L., Roh W., Reuben A., Cooper Z.A., Spencer C.N., Prieto P.A., Miller J.P., Bassett R.L., Gopalakrishnan V., Wani K. (2016). Analysis of immune signatures in longitudinal tumor samples yields insight into biomarkers of response and mechanisms of resistance to immune checkpoint blockade. Cancer Discov..

[73] Hamid O., Schmidt H., Nissan A., Ridolfi L., Aamdal S., Hansson J., Guida M., Hyams D.M., Gómez H., Bastholt L. (2011). A prospective phase II trial exploring the association between tumor microenvironment biomarkers and clinical activity of ipilimumab in advanced melanoma. J. Transl. Med..

[74] Tumeh P.C., Harview C.L., Yearley J.H., Shintaku I.P., Taylor E.J., Robert L., Chmielowski B., Spasic M., Henry G., Ciobanu V. (2014). PD-1 blockade induces responses by inhibiting adaptive immune resistance. Nature.

[75] Le D.T., Durham J.N., Smith K.N., Wang H., Bartlett B.R., Aulakh L.K., Lu S., Kemberling H., Wilt C., Luber B.S. (2017). Mismatch repair deficiency predicts response of solid tumors to PD-1 blockade. Science.

[76] Hugo W., Zaretsky J.M., Sun L., Song C., Moreno B.H., Hu-Lieskovan S., Berent-Maoz B., Pang J., Chmielowski B., Cherry G. (2017). Genomic and Transcriptomic Features of Response to Anti-PD-1 Therapy in Metastatic Melanoma. Cell.

[77] Gettinger S.N., Wurtz A., Goldberg S.B., Rimm D., Schalper K., Kaech S., Kavathas P., Chiang A., Lilenbaum R., Zelterman D. (2018). Clinical Features and Management of Acquired Resistance to PD-1 Axis Inhibitors in 26 Patients With Advanced Non-Small Cell Lung Cancer. J. Thorac. Oncol..

[78] Aslan K., Turco V., Blobner J., Sonner J.K., Liuzzi A.R., Nunez N.G., De Feo D., Kickingereder P., Fischer M., Green E. (2020). Heterogeneity of response to immune checkpoint blockade in hypermutated experimental gliomas. Nat. Commun..

[79] Williams J.B., Li S., Higgs E.F., Cabanov A., Wang X., Huang H., Gajewski T.F. (2020). Tumor heterogeneity and clonal cooperation influence the immune selection of IFN-gamma-signaling mutant cancer cells. Nat. Commun..

[80] Zitvogel L., Ma Y., Raoult D., Kroemer G., Gajewski T.F. (2018). The microbiome in cancer immunotherapy: Diagnostic tools and therapeutic strategies. Science.

[81] Gopalakrishnan V., Spencer C.N., Nezi L., Reuben A., Andrews M.C., Karpinets T.V., Prieto P.A., Vicente D., Hoffman K., Wei S.C. (2018). Gut microbiome modulates response to anti-PD-1 immunotherapy in melanoma patients. Science.

[82] Matson V., Fessler J., Bao R., Chongsuwat T., Zha Y., Alegre M.L., Luke J.J., Gajewski T.F. (2018). The commensal microbiome is associated with anti-PD-1 efficacy in metastatic melanoma patients. Science.

[83] Chen G., Huang A.C., Zhang W., Zhang G., Wu M., Xu W., Yu Z., Yang J., Wang B., Sun H. (2018). Exosomal PD-L1 contributes to immunosuppression and is associated with anti-PD-1 response. Nature.

[84] Poggio M., Hu T., Pai C.C., Chu B., Belair C.D., Chang A., Montabana E., Lang U.E., Fu Q., Fong L. (2019). Suppression of Exosomal PD-L1 Induces Systemic Anti-tumor Immunity and Memory. Cell.

